# Clinical Relevance of Liver Kinase B1(LKB1) Protein and Gene Expression in Breast Cancer

**DOI:** 10.1038/srep21374

**Published:** 2016-02-15

**Authors:** I-Chun Chen, Yuan-Ching Chang, Yen-Shen Lu, Kuei-Pin Chung, Chiun-Sheng Huang, Tzu-Pin Lu, Wen-Hung Kuo, Ming-Yang Wang, Kuan-Ting Kuo, Pei-Fang Wu, Tsu-Hsin Hsueh, Chen-Yang Shen, Ching-Hung Lin, Ann-Lii Cheng

**Affiliations:** 1 Department of Oncology, National Taiwan University Hospital; 2 Graduate Institute of Oncology, National Taiwan University; 3 Department of Surgery, Mackay Memorial Hospital; 4 Department of Internal Medicine, National Taiwan University Hospital; 5 Departent of Laboratory Medicine, National Taiwan University Hospital; 6 Department of Surgery, National Taiwan University Hospital; 7 Department of Public Health, National Taiwan University; 8 Department of Pathology, National Taiwan University Hospital; 9 Department of Medical Research, National Taiwan University Hospital; 10 Graduate Institute of Life Sciences, National Defense Medical Center, Taipei; 11 Institute of Biomedical Sciences, Academia Sinica, Taipei, Taiwan; 12 College of Public Health, China Medical University, Taichung, Taiwan; 13 Oncology Center, National Taiwan University Hospital Hsin-Chu Branch.

## Abstract

Liver kinase B1 (LKB1) is a tumor suppressor, and its loss might lead to activation of the mammalian target of rapamycin (mTOR) and tumorigenesis. This study aimed to determine the clinical relevance of LKB1 gene and protein expression in breast cancer patients. LKB1 protein expression was evaluated using immunohistochemistry in tumors from early breast cancer patients in two Taiwanese medical centers. Data on *LKB1* gene expression were obtained from the Molecular Taxonomy of Breast Cancer International Consortium (METABRIC) data set. The correlations between LKB1 expression, clinicopathologic factors, and patient outcome were analyzed. LKB1 expression was significantly associated with estrogen receptor (ER) expression in 2 of the 4 cohorts, but not with other clinicopathologic factors. LKB1 expression was not a predictor for relapse-free survival, overall survival (OS), or breast cancer-specific survival. In a subgroup analysis of the two Taiwanese cohorts, high LKB1 protein expression was predictive of high OS in human epidermal growth factor receptor 2 (HER2)-positive breast cancer patients (*P* = 0.013). Our study results indicate that LKB1 expression is not prognostic in the whole population of breast cancer patients, but it is a potential predictor of OS in the subset of HER2-positive patients

Liver kinase B1 (LKB1) is a tumor suppressor gene identified in hereditary Peutz-Jeghers syndrome^1^. It is involved in the control of metabolism^2^ and tumorigenesis^3^. When LKB1 interacts with AMP-activated protein kinase (AMPK), it abrogates signal transduction through the mammalian target of rapamycin (mTOR) to inhibit cell growth^4^, thus functioning as a tumor suppressor^2^. In previous studies on breast cancer, reduced LKB1 expression was associated with increased metastatic and invasive potential^5^. Therefore, LKB1-related pathways might provide potential targets for mitigating the invasive and metastatic characteristics of breast cancer.

A previous study evaluated LKB1 expression in metastatic estrogen receptor (ER)-positive breast cancer samples (n = 55) from the TAMRAD trial^6^,and observed that low LKB1 expression was predictive of everolimus efficacy. This study is the only human study to indicate the predictive value of LKB1 in targeted therapy for breast cancer. Other studies evaluating the role of LKB1 in breast cancer patients have provided inconsistent results. Two studies have evaluated the prognostic value of LKB1 in breast cancer patients. In Shen *et al.*^7^, low LKB1 expression was associated with low relapse-free survival (RFS) and overall survival (OS) in 116 breast cancer patients. By contrast, in Bouchekioua-Bouzaghou *et al.*^8^, high cytoplasmic LKB1 expression was associated with low disease-free survival (DFS) in a cohort of 154 breast cancer patients. In other studies, LKB1 status correlated with ER but in opposite directions. In a study on MCF-7 human breast cancer cells^9^, ERα downregulated LKB1 expression by regulating its promoter regions. In 2 human breast cancer studies, ER expression was associated with low LKB1^8^ and high LKB1^5^ expression. However, these studies have had relatively small samples (n = 154 and 80, respectively), thus limiting the ability to stratify patients into prognostic groups for LKB1 assessment. Nevertheless, the results have indicated that LKB1 might play a key role in breast cancer biology; therefore, its relationships with survival and ER status warrant further investigation.

In this study, we investigated the role of LKB1 in breast cancer by evaluating LKB1 protein expression in 2 Asian cohorts using immunohistochemistry (IHC) stain, and by evaluating *LKB1* gene expression in microarray data sets from 2 Western cohorts^9^. We analyzed the associations of LKB1 protein and gene expression with clinicopathologic factors, such as ER and human epidermal growth factor receptor 2 (HER2) statuses, and survival. We assessed the predictive value of LKB1 for survival outcomes according to various risk and prognostic groups.

## Results

### Patient characteristics

The demographic data of the 4 study cohorts are listed in Supplementary Table 1. We collected 730 and 307 archival breast cancer patient samples from the NTUH and MMH cohorts, from which LKB1 IHC analysis results were evaluable in 600 and 290 samples, respectively. The METABRIC discovery and validation cohorts consisted of 997 and 995 patients, respectively. The numbers of stage I–III patients from the 4 cohorts included in final analyses of clinicopathologic factors and survival status were, sequentially, 569 (NTUH), 277 (MMH), 988 (METABRIC discovery), and 975 (METABRIC validation).

The median ages of the NTUH and the MMH cohorts were 48.0 and 54.0 years, respectively; those of the METABRIC discovery and validation cohorts were 61.3 and 62.6 years, respectively. The major histological subtypes were invasive ductal carcinoma (80.7–94.9%) and invasive lobular carcinoma (1.6–12.4%). Of the cancer stages, stage II was dominant in all 4 cohorts. The NTUH and MMH cohorts contained higher numbers of HER2-positive patients than the METABRIC cohorts did, whereas the METABRIC cohorts contained higher numbers of ER-positive patients than the NTUH and MMH cohorts did.

### Correlation between liver kinase B1 protein expression and clinicopathologic factors or survival

**Figure 1:**
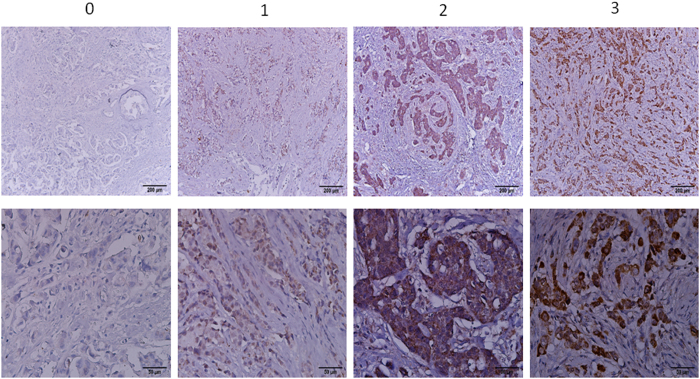
LKB1 IHC stains: NTUH cohort Immunohistochemical staining of LKB1 in breast cancer patients in NTUH cohort.

Figure 1 and Supplementary Fig. 1 showed the representative LKB1 IHC staining (scored as 0, 1, 2, and 3) in the NTUH and the MMH cohorts, respectively. LKB1 expression was high in 71.7% and 68.2% of the stage I–III breast cancer patients from the NTUH and the MMH cohorts (Table 1). Low LKB1 protein expression was significantly associated with high ER positivity (*P* = 0.002) and high PR positivity (*P* = 0.018) in the MMH cohort, but not in the NTUH cohort. LKB1 protein expression was not associated with clinicopathologic factors such as HER2 status, tumor size, lymph node status, stage, grade, and menopause in the NTUH and MMH cohorts.

**Figure 2:**
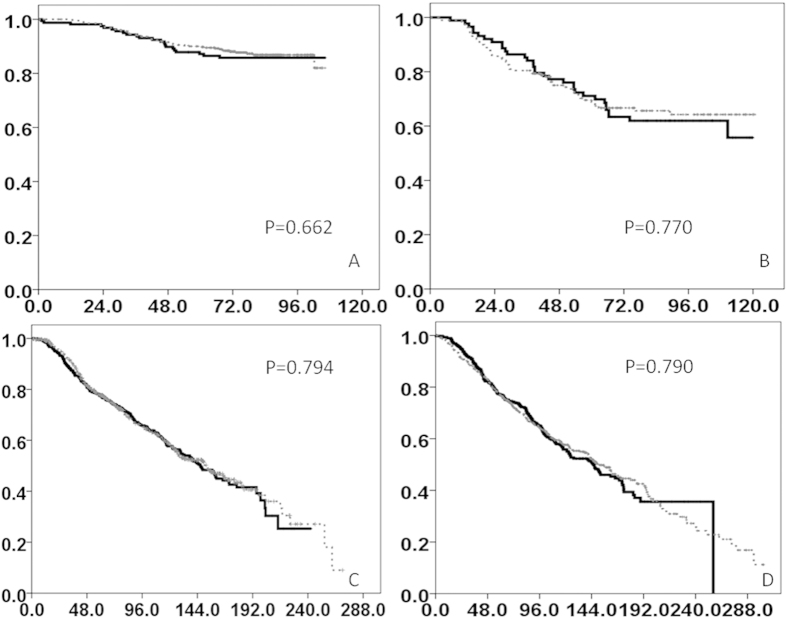
OS The overall survival is shown in months. (**A**)NTUH cohort (**B**) MMH cohort (**C**) Discovery cohort (**D**) Validation cohort (Dotted line: LKB1 high, solid line: LKB1 low).

**Figure 3:**
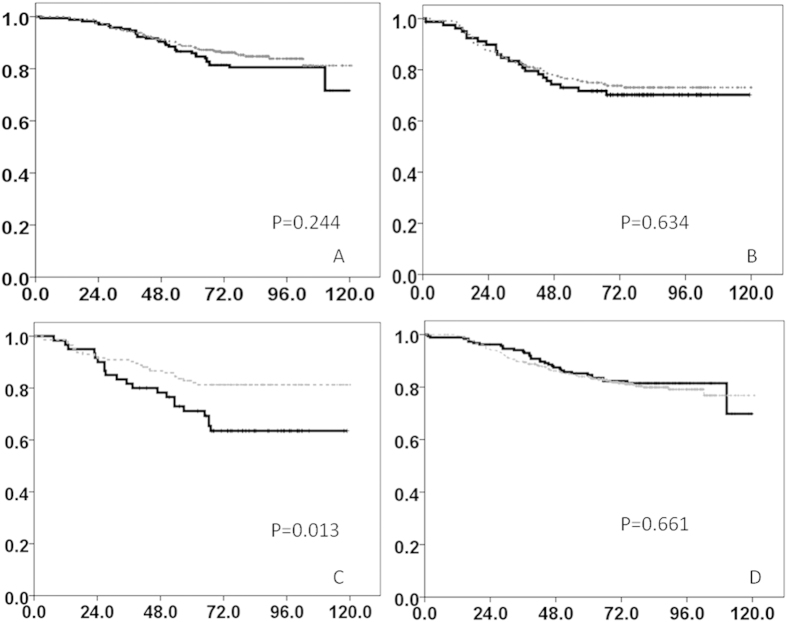
OS of NTUH and MMH cohorts in subgroups. (**A**) ER positive (**B**) ER negative (**C**) HER2 positive (**D**) HER2 negative (Dotted line:LKB1 high, solid line:LKB1 low).

In the NTUH and MMH cohorts, the median duration of follow-up was 77.8 and 62.6 months, respectively. LKB1 protein expression was not predictive of RFS or OS (Fig. 2A,B) in the 2 cohorts. To minimize the effects of sample size on predictive value in survival analyses, we combined the patients from the NTUH and MMH cohorts in a Cox regression model analysis. Table 2 lists the major prognostic factors for OS in the 2 cohorts: ER positivity (HR = 0.523, *P* < 0.0001), tumor size (*P* < 0.0001), lymph node status (*P* < 0.0001), and menopause (HR = 1.631, *P* = 0.006). ER positivity (HR = 0.644, *P* = 0.008), tumor size (*P* < 0.001), lymph node status (*P* < 0.001), and grade (*P* = 0.014) were predictive of RFS in both cohorts (Supplementary Table 2). For subgroup analyses based on ER and HER2 statuses, we stratified the patients from the NTUH and MMH cohorts into ER-positive/negative and HER2-positive/negative groups (Fig. 3). High LKB1 protein expression was prognostic for high OS in the HER2-positive subgroup (*P* = 0.013) (Fig. 3C), but not in the remaining 3 subgroups (Fig. 3A,B,D).

### *LKB1* gene expression, clinicopathologic factors, and survival

We divided the 2 METABRIC cohorts into low and high *LKB1* gene expression groups. High ER positivity was associated with low *LKB1* gene expression in the METABRIC discovery cohort (*P* = 0.039) (Table 1), but not in the validation cohort. Other clinicopathologic factors were not associated with *LKB1* gene expression in the METABRIC discovery and validation cohorts.

The median duration of follow-up in the METABRIC discovery and validation cohorts was 83.8 and 87.8 months, respectively. When we analyzed OS by using a Cox regression model, *LKB1* status was not predictive of OS in all stage I–III patients from the 2 cohorts (HR = 0.937 and 1.024, *P* = 0.512 and 0.816, respectively) (Table 2, Fig. 2C,D). We observed no difference in OS between the high *LKB1* and low *LKB1* groups (Fig. 3). The major predictors for high OS (Table 2) and high BSS (Table 3) in the 2 METABRIC cohorts were small tumor size and low lymph node involvement. Menopause was predictive of low OS in both cohorts, but not predictive of BSS. ER positivity (HR = 0.770, *P* = 0.050) and HER2 status (HR = 1.479, *P* = 0.008) were predictive of OS in the discovery cohort, but not in the validation cohort. In subgroup analyses, *LKB1* gene expression was nonsignificantly associated with OS and BSS in the ER-positive/negative and HER-positive/negative subgroups (Supplementary Figs 2–5).

### Surrogate makers of LKB1 catalytic function

The catalytic function of LKB1 could not be directly analyzed by IHC in the formalin fixed paraffin embedded slides or by gene expressions. We tested phosphorylated AMP- activated protein kinase (pAMPK) and phosphorylated acetyl-CoA carboxylase(pACC) status as potential surrogate markers of LKB1 catalytic function in breast cancer. We randomly selected 108 tumor samples from the NTUH cohort and conducted IHC for pAMPK and pACC. The representative figures for pAMPK and pACC staining were shown in Supplementary Fig. 7 and their correlations with LKB1 expression were shown in Supplementary Table 3. LKB1 expression was positively associated with pACC expression (p = 0.0003), but it was not associated with pAMPK expression (p = 0.700). Neither pACC nor pAMPK expression was associated with other clinical factors assessed in this study (data not shown).

## Discussion

Our study evaluated 2809 stage I–III breast cancer patients in 4 cohorts to investigate the relationships between LKB1 expression and clinicopathologic factors or patient outcome. Our results indicated nonsignificant associations between LKB1 protein and gene expression and OS, BSS, or RFS in the stage I–III breast cancer patients. However, in subgroup analyses, high LKB1 protein expression was associated with high OS in the HER2-positive population from the 2 Asian cohorts.

LKB1 expression correlated with ER positivity in 2 of the study cohorts but in opposite directions. Consistent with Linher-Melville *et al.*^10^, high ER positivity was associated with low LKB1 expression in the MMH cohort. Linher-Melville *et al.* evaluated LKB1 expression a MCF-7 cell line, and observed that ERα is a downregulator of *LKB1* gene expression. Thus, when ER is highly expressed, it leads to low *LKB1* expression and low LKB1 protein expression. In the METABRIC discovery cohort, high *LKB1* gene expression was associated with high ER positivity. This observation might be explained by the results from a previous study on LKB1 and ERα signaling^11^ that evaluated the role of LKB1 as a coactivator for ERα according to its catalytic function in the nucleus. When the authors used siRNA to knock down LKB1, ERα activity was downregulated, indicating that LKB1 and ERα might interact reciprocally as a control mechanism. In addition, LKB1 might be associated with its regulator ER in some, but not all, conditions, which might explain the inconsistencies in results on the relationship between LKB1 and ER among our and previous studies. In Brown *et al.*^12^, they have demonstrated that ERα would bind to LKB1 promoter region in the presence of estradiol in MCF7 cells. This provides an explanatory mechanism for potentially low LKB1 in ER positive breast cancer. Individual differences in the control mechanisms for LKB1 and ER in breast cancer patients might also exist, but we could not evaluate the possible differences in this study.

In addition to the presence of LKB1 in ERα functional modulation, LKB1 catalytic function also lead to enhanced transactivation of ERα^11^. This catalytic function might be lost after point mutation in LKB1 or loss of LKB1^13^ resulting in altered ERα function. LKB1 is also a pivotal kinase to control AMPK subfamily by catalyzing AMPK phosphorylation^14^ and therefore pAMPK and its downstream pACC might be surrogate markers for functional LKB1. In the present study, the pAMPK status was not associated with LKB1 expression. A prior study showed that AMPK can also be phosphorylated by calmodulin-dependent protein kinase kinase-beta in LKB1 deficient conditions^15^. Therefore, the association of pAMPK with LKB1 might not be as evident as pACC in breast cancer. In contrast, the positive association between LKB1 and pACC suggested that pACC could be a surrogate marker for LKB1 catalytic function in breast cancer. This finding is consistent with the study by Carretero *et al.* that *LKB1*-negative primary lung adenocarcinoma had very low level of pACC protein^16^.

In our subgroup analyses, we stratified the 4 cohorts into 4 groups according to ER and HER2, based on the prognostic^17^,^18^,^19^,^20^,^21^ and treatment grouping of breast cancer patients. High LKB1 protein expression was predictive of high OS in the HER2-positive population in the 2 Asian cohorts. In previous LKB1 human studies, HER2-positive patients were not well described. Shen *et al.*^7^,^8^ did not specify the percentage of HER2-positive patients. In Bouchekioua-Bouzaghou *et al.*^8^, the HER2-positive population was between 7.7% and 15%. A biomarker analysis from the FinHER trial^22^ detected one case of LKB1 mutation in 687 genotyped HER2-positive patient tumors. However, because the study focused on somatic mutational status in HER2-positive patients, the presence or loss of LKB1 was not analyzed.

Although previous human studies did not establish an association between HER2 and LKB1, loss of *LKB1* led to reduced elapsed time for HER2-mediated tumorigenesis^2^,^23^ in previous studies on *LKB1* knockout mice. The HER2-positive tumor in *LKB1*^−/−^ mice has an altered metabolic pathway, suggesting that loss of LKB1 might be an indicator of hyperactive mTOR in HER2-positive breast cancer. Andrade-Vieira *et al.*^24^ treated HER2-positive tumor samples from *LKB1*^−/−^ mice with an mTOR inhibitor and 2-deoxyglucose and observed favorable tumor control. In the BOLERO-3 study^25^, adding the mTOR inhibitor everolimus to trastuzumab and vinorelbine increased progression-free survival in HER2-positive trastuzumab-resistant breast cancer patients. Our subgroup analysis result of an association between high LKB1 and high OS in HER2 patients might support these BOLERO-3 results. However, this association requires further validation in a large HER2-enriched cohort.

The major differences between our and the described previous human LKB1 studies are sample size, stratification, definition of LKB1, and duration of follow-up. Our study evaluated 2809 patients in 4 cohorts, which is an approximately 24-fold and 18-fold larger sample than the samples evaluated in Shen *et al.*^7^ and Bouchekioua-Bouzaghou *et al.*^8^, respectively. In these 2 studies, stratification of the patients according to biological factors and treatment was not feasible because of limited patient numbers. In our study, when we analyzed the entire population, LKB1 was not prognostic. However, when we stratified the patients according to HER2 status, high LKB1 protein expression was predictive of high OS in the HER2-positive subgroup. LKB1 might thus play differing biological roles in different breast cancer groups. Our and the described human LKB1 studies also differ in the methods used for LKB1 analysis. Shen *et al.*^7^ evaluated LKB1 expression in 116 breast cancer patients by using Western blot analysis with a LKB1 polyclonal antibody. The authors defined low or high LKB1 expression according to the levels of LKB1 expression in the liver. When the ratio of tumor LKB1:liver LKB1 was lower than 0.5, it was defined as low LKB1 expression. According to this definition, the authors categorized 34.2% of the patients (38 of 111 evaluable LKB1 patients) as low LKB1 expression. Although our methods for defining LKB1 expression differed from those used in the described study, the percentages of low LKB1 expression patients in our NTUH and MMH cohorts were comparable. Bouchekioua-Bouzaghou *et al.*^8^ evaluated LKB1 in 154 breast cancer patients by using IHC analysis and observed that low cytoplasmic LKB1 expression was associated with ER positivity. Our IHC methods for defining LKB1 are consistent with the methods used in the study. In the 2 METABRIC cohorts, we defined LKB1 expression based on levels of mRNA expression. The final methodological difference between our and previous studies is the duration of follow-up. A sufficient follow-up is mandatory in outcome studies to observe survival events. In our study, the median duration of follow-up was between 62.6 and 87.8 months in the 4 cohorts. In Shen *et al.*^7^, the median duration of follow-up was 44.5 months. In Bouchekioua-Bouzaghou *et al.*^8^, the median duration of follow-up was not mentioned. Therefore, the strengths of our study include its relatively large cohort and long duration of follow-up, which enable the associations between LKB1 and survival status to be more accurately assessed than in the described small-scale studies.

Our study has limitations. First, when the median level of mRNA expression is used as a cutoff for discriminating high from low LKB1 status, a non-normal distribution can lead to statistical biases. However, we analyzed LKB1 expression and survival by dividing LKB1 expression into 3 or 4 groups (Supplementary Fig. 5), and our results indicated no correlation between LKB1 and survival. Second, the two Asian cohorts are retrospectively collected samples which will need larger validation cohorts to confirm our observation^6^. The prospectively collected METABRIC cohorts served as one of the good validation resource, but further mechanistic or prospective studies are complimentary to confirm the phenomena discovered in this study. The last limitation is that relapse status was unknown in the 2 Western cohorts. Therefore, we analyzed only LKB1 expression and RFS in the 2 Asian cohorts. However, the samples from the 2 Asian cohorts were considerably larger than those from previous studies and should provide sufficient information for RFS analysis.

## Conclusion

LKB1 might be a prognostic factor in HER2-positive breast cancer patients, but is not consistently associated with other clinicopathologic variables. Our exploratory findings from subgroup analyses warrant further validation in a large HER2-enriched breast cancer population. Our study results might indicate a potential additional targetable mechanism for treatment of HER2-positive breast cancer patients.

## Materials and Methods

### Patient cohorts

Four cohorts were evaluated in this study. The first cohort consisted of breast cancer patients diagnosed between 2004 and 2007 at National Taiwan University Hospital (NTUH cohort), from whom archival paraffin slides were collected for LKB1 IHC analysis. The second cohort was composed of breast cancer patients diagnosed between 2004 and 2010 at Mackay Memorial Hospital (MMH cohort). Tissue microarray slides from the MMH cohort were obtained for LKB1 IHC analysis. The human sample collection in National Taiwan University Hospital (NTUH) and Mackay Memorial Hospital (MMH) are approved by the institutional research committees in both institutions. The informed consents were obtained from all of the subjects enrolled in NTUH and MMH. All the methods carried out in this study are in accordance with the approved protocols by the Institutional Review Board of NTUH. The third and fourth cohorts were from the publicly available Molecular Taxonomy of Breast Cancer International Consortium (METABRIC) data sets (discovery and validation cohorts). Their raw data were analyzed for *LKB1* expression. Supplementary Table 1 lists the demographic data of the 4 cohorts.

### Immunohistochemical analysis of liver kinase B1, phosphorylated AMP-activated protein kinase, and phosphorylated acetyl-CoA carboxylase expression

Rabbit antihuman LKB1 antibody (ab58786) was purchased from Abcam (Cambridge MA, USA). Phospho-AMPKα (Thr172) (40H9) rabbit monoclonal antibody (#2535) and phospho-Acetyl-CoA Carboxylase (Ser79) (D7D11) rabbit monoclonal antibody (#11818) were purchased from Cell Signaling (Danvers MA, USA). Four-micrometer slides from formalin-fixed paraffin blocks were deparaffinized using xylene and rehydrated using serial gradient ethanol. The anti-LKB1 antibody was diluted to 1:100. The remaining standard staining procedure was performed according to manufacturer manual. Omission of the primary antibody incubation was served as negative control for every batch of LKB1 staining, and normal breast epithelial cells on the slides were served as internal positive control^26^.

LKB1 protein expression was categorized into 4 groups according to the intensity and extent of IHC staining, using the standards described in Bouchekioua-Bouzaghou *et al.*^8^. When the cells showed no positivity or <10% positive staining, they were scored as 0 and 1, respectively. When 10%–50% or >50% positive staining was observed, they were scored as 2 and 3, respectively. Low LKB1 expression was defined as a score of 0 or 1, and high LKB1 expression was defined as a score of 2 or 3.

### *LKB1* gene expression from the Molecular Taxonomy of Breast Cancer International Consortium database

Data on *LKB1* gene expression were available online for all patients in the METABRIC cohorts. Because the recurrence status was not recorded in the original METABRIC database, it was not analyzed in these 2 cohorts. In the METABRIC database, *LKB1* gene expression was normalized to the median expression level in the data set. *LKB1* gene expression levels higher than the median *LKB1* gene expression level were categorized as high, whereas those lower than the median *LKB1* gene expression level were categorized as low.

### Statistical analysis

Stage I–III patients from the 4 cohorts were included in analyses of the correlations between LKB1 status and clinicopathologic factors and outcomes. A crosstable analysis was applied to correlate LKB1 status with age, menopause, ER status, progesterone receptor (PR) status, HER2 status, stage, grade, and OS in the 4 cohorts. RFS was analyzed in the NTUH and the MMH cohorts. To determine the influence of sample size on the NTUH and MMH cohorts, a Cox regression model analysis for OS and RFS was performed using the combined data from the 2 cohorts. Breast cancer-specific survival (BSS) was analyzed in the 2 METABRIC cohorts. Patients were stratified according to ER and HER2 status for subgroup analysis of OS, RFS, and BSS. A chi-squared test was applied to determine significance.

## Additional Information

**How to cite this article**: Chen, I.-C. *et al.* Clinical Relevance of Liver Kinase B1(LKB1) Protein and Gene Expression in Breast Cancer. *Sci. Rep.* **6**, 21374; doi: 10.1038/srep21374 (2016).

## Supplementary Material

Supplementary Files

## Figures and Tables

**Table 1:** LKB1 and Clinicopathologic Factors.

	NTUH			MMH		
	LKB1 High	LKB1 Low	Total	LKB1 High	LKB1 Low	Total
Patient Number	408(71.7%)	161(28.3%)	569(100%)	189(68.2%)	88(31.8%)	277(100%)
ER						
Negative	129(31.6%)	54(33.5%)	183(32.2%)	94(49.7%)	26(29.5%)	120(43.3%)
Positive	279(68.4%)	107(66.5%)	386(67.8%)	95(50.3%)	62(70.5%)	157(56.7%)
p	0.691			0.002*		
PR						
Negative	231(56.6%)	94(58.3%)	325(57.1%)	122(64.6%)	43(48.9%)	165(59.6%)
Positive	177(43.4%)	67(41.6%)	244(42.9%)	67(35.4%)	45(51.1%)	112(40.4%)
p	0.708			0.018*		
HER2						
Negative	319(78.2%)	127(78.9%)	446(78.4%)	131(70.8%)	61(70.1%)	87(32.0%)
Positive	89(21.8%)	34(21.1%)	123(21.6%)	54(29.2%)	26(29.9%)	185(68.0%)
p	0.910			1.000		
Menopause						
Premenopause	222(54.4%)	100(62.1%)	322(56.6%)	72(38.1%)	36(40.9%)	108(39.0%)
Postmenopause	186(45.6%)	61(37.9%)	247(43.4%)	117(61.9%)	52(59.1%)	169(61.0%)
p	0.110			0.692		
p	0.575			0.691		
T						
1	183(44.9%)	71(44.1%)	254(44.8%)	27(14.3%)	22(25.0%)	49(17.7%)
2	189(46.3%)	74(46.0%)	263(46.4%)	147(77.8%)	60(68.2%)	207(74.7%)
3/4	34(8.3%)	16(9.9%)	50(8.8%)	15(7.9%)	6(6.8%)	21(7.6%)
P	0.922			0.094		
N						
0	253(62.5%)	91(57.6%)	344(61.0%)	83(43.9%)	32(36.4%)	115(41.5%)
1	111(27.4%)	54(34.2%)	165(29.3%)	49(25.9%)	25(28.4%)	74(26.7%)
2	25(6.2%)	8(5.1%)	33(5.9%)	33(17.5%)	12(13.6%)	45(16.2%)
3	16(4.0%)	5(3.2%)	21(3.7%)	24(12.7%)	19(21.6%)	43(15.5%)
P	0.551		0.207			
Stage						
1	139(34.1%)	53(32.9%)	192(33.7%)	19(10.1%)	12(13.6%)	31(11.2%)
2	212(52.0%)	85(52.8%)	297(52.2%)	104(55.0%)	40(45.5%)	144(52%)
3	57(14.0%)	23(14.3%)	80(14.1%)	66(34.9%)	36(40.9%)	102(36.8%)
P	0.966			0.312		
Grade						
1	81(19.9%)	25(15.5%)		12(6.3%)	2(2.4%)	14(5.1%)
2	212(52.0%)	86(53.4%)		99(52.4%)	55(64.7%)	154(56.2%)
3	99(24.3%)	40(24.8%)		78(41.3%)	28(32.9%)	106(38.7%)
Unknown	16(3.9%)	10(6.2%)		0	0	
p	0.467			0.107		
		
	**METBRIC Discovery Cohort**			**METBRIC Validation Cohort**		
	**LKB1 High**	**LKB1 Low**	**Total**	**LKB1 High**	**LKB1 Low**	**Total**
Patient Number	**494(50.0%)**	**494(50.0%)**	**988(100%)**	**487(50.0%)**	**488(50.0%)**	**975(100%)**
ER						
Negative	86(17.4%)	113(22.9%)	199(20.1%)	124(26.2%)	131(27.2%)	255(26.7%)
Positive	408(82.6%)	381(77.1%)	789(79.9%)	350(73.8%)	350(73.8%)	700(73.3%)
p	0.039*			0.715		
PR						
Negative	226(45.7%)	242(49.0%)	468(47.4%)	223(47.0%)	224(46.6%)	447(46.8%)
Positive	268(54.3%)	252(51.0%)	520(52.6%)	251(53.0%)	257(53.4%)	508(53.2%)
p	0.339			0.897		
HER2						
Negative	440(89.1%)	432(87.4%)	494(88.2%)	416(87.8%)	419(85.9%)	829(86.8%)
Positive	54(10.9%)	62(12.6%)	116(11.7%)	58(12.2%)	68(14.1%)	126(13.2%)
p	0.489			0.391		
Menopause						
Premenopause	106(21.7%)	129(26.4%)	235(24.1%)	91(19.2%)	100(20.8%)	191(20.0%)
Postmenopause	382(78.3%)	360(73.6%)	742(75.9%)	383(80.8%)	381(79.2%)	764(80.0%)
p	0.100			0.571		
T						
1	205(41.5%)	230(46.6%)	435(44.0%)	208(44.3%)	207(43.1%)	415(43.7%)
2	263(53.2%)	244(49.4%)	507(51.3%)	238(50.6%)	244(50.8%)	482(50.7%)
3/4	26(5.3%)	20(4.0%)	46(4.7%)	24(5.1%)	29(6.0%)	53(5.6%)
p	0.231			0.801		
N						
0	259(52.4%)	253(51.2%)	512(51.8%)	260(55.0%)	238(49.7%)	498(52.3%)
1	156(31.6%)	161(32.6%)	317(32.1%)	136(28.8%)	165(34.4%)	301(31.6%)
2	61(12.3%)	64(13.0%)	125(12.7%)	50(10.6%)	49(10.2%)	99(10.4%)
3	18(3.6%)	16(3.2%)	34(3.4%)	27(5.7%)	27(5.6%)	54(5.7%)
p	0.953			0.291		
Stage						
1	141(28.5%)	155(31.4%)	296(30.0%)	133(28.1%)	124(25.8%)	257(26.9%)
2	265(53.6%)	254(51.4%)	519(52.5%)	255(53.8%)	269(55.9%)	524(54.9%)
3	88(17.8%)	85(17.2%)	173(17.5%)	85(18.1%)	88(18.3%)	174(18.2%)
p	0.623			0.719		
Grade						
1	35(3.5%)	37(3.7%)	72(7.2%)	46(9.7%)	49(10.2%)	95(9.9%)
2	212(21.5%)	198(20.0%)	410(41.5%)	182(38.4%)	168(34.9%)	350(36.6%)
3	247(25.0%)	259(26.2%)	506(51.2%)	216(45.6%)	224(46.6%)	440(46.1%)
Unknown	0	0		30(6.3%)	40(8.3%)	70(7.3%)
P	0.534			0.535		

**Table 2:** Cox Regression Model: Overall survival.

	NTUH			MMH			NTUH/MMH Summary Analysis		
	HR	95%CI	P	HR	95%CI	P	HR	95%CI	P
LKB1(IHC)			0.321			0.823			0.419
Negative	1.000			1.000			1.000		
Positive	0.766	0.453–1.296		1.054	0.665–1.671		0.868	0.615–1.224	0.419
ER			0.001*			0.064			<0.0001*
Negative	1.000			1.000			1.000		
Positive	0.369	0.210–0.648		0.620	0.374–1.028		0.523	0.365–0.748	
HER2			0.233			0.247			0.211
Negative	1.000			1.000			1.000		
Positive	0.690	0.375–1.269		0.753	0.466–1.217		0.790	0.547–1.142	
T			0.193			<0.0001*			<0.0001*
T1	1.000			1.000			1.000		
T2	1.657	0.910–3.016	0.098	0.732	0.424–1.263	0.262	1.169	0.785–1.742	0.442
T3	2.114	0.838–5.334	0.113	1.901	0.820–4.404	0.134	1.716	0.951–3.096	0.073
T4	2.881	0.811–10.232	0.102	3.499	1.502–8.150	0.004	3.976	2.038–7.757	<0.0001
N			0.007*			<0.0001*			<0.0001*
N0	1.000			1.000			1.000		
N1	1.349	0.742–2.450	0.326	2.325	1.231–4.390	0.009	1.744	1.139–2.671	0.011
N2	3.088	1.444–6.601	0.004	4.186	2.130–8.226	<0.0001	4.587	2.895–7.269	<0.0001
N3	3.292	1.356–7.991	0.008	5.329	2.730–10.399	<0.0001	6.004	3.712–9.711	<0.0001
Grade			0.809			0.361			0.105
Grade 1	1.000			1.000			1.000		
Grade 2	1.257	0.574–2.752	0.567	2.692	0.630–11.510	0.182	1.955	1.005–3.805	0.048
Grade 3	1.327	0.557–3.161	0.523	2.946	0.668–13.002	0.154	2.127	1.051–4.306	0.036
Menopause			0.083			0.105			0.006*
Negative	1.000			1.000			1.000		
Positive	1.586	0.941–2.674		1.501	0.918–2.453		1.631	1.149–2.317	
									
	**METBRIC Discovery Cohort**			**METBRIC Validation Cohort**					
	**HR**	**95%CI**	**P**	**HR**	**95%CI**	**P**			
*LKB1*(mRNA)			0.512			0.816			
Negative	1.000			1.000					
Positive	0.937	0.772–1.138	0.512	1.024	0.839–1.250	0.816			
ER			0.050*			0.080			
Negative	1.000			1.000					
Positive	0.770	0.593–1.000		0.794	0.613–1.028				
HER2			0.008*			0.521			
Negative	1.000			1.000					
Positive	1.479	1.110–1.972		1.101	0.821–1.476				
T			0.007*			<0.0001*			
T1	1.000			1.000					
T2	1.334	1.076–1.653	0.009	1.701	1.369–2.114	<0.0001			
T3	1.798	1.129–2.864	0.013	2.381	1.600–3.543	<0.0001			
N			<0.0001*			<0.0001*			
N0	1.000			1.000					
N1	1.307	1.038–1.647	0.023	1.385	1.102–1.740	0.005			
N2	2.112	1.594–2.799	<0.0001	2.060	1.508–2.814	<0.0001			
N3	4.343	2.682–7.032	<0.0001	3.433	2.370–4.973	<0.0001			
Grade			0.713			0.240			
Grade 1	1.000			1.000					
Grade 2	1.044	0.664–1.640	0.852	1.381	0.926–2.060	0.113			
Grade 3	1.134	0.719–1.788	0.590	1.411	0.937–2.124	0.099			
Menopause			0.001*			0.008*			
Negative	1.000			1.000					
Positive	1.543	1.194–1.994		1.443	1.099–1.894				

**Table 3:** Cox Regression Model: Breast Cancer Specific Survival.

	METBRIC Discovery Cohort			METBRIC Validation Cohort		
	HR	95%CI	P	HR	95%CI	P
*LKB1*(mRNA)			0.856			0.282
Negative	1.000			1.000		
Positive	0.977	0.761–1.255		0.865	0.665–1.126	
ER			0.054			0.021*
Negative	1.000			1.000		
Positive	0.726	0.524–1.005		0.680	0.490–0.943	
HER2			0.003*			0.056
Negative	1.000			1.000		
Positive	1.669	1.186–2.348		1.393	0.991–1.959	
T			0.049*			<0.0001*
T1	1.000			1.000		
T2	1.316	0.991–1.747	0.058	1.709	1.271–2.299	<0.0001
T3	1.841	1.051–3.223	0.033	2.287	1.390–3.761	0.001
N			<0.0001*			<0.0001*
N0	1.000			1.000		
N1	1.628	1.201–2.208	0.002	1.787	1.299–2.459	<0.0001
N2	2.771	1.945–3.948	<0.0001	3.223	2.163–4.803	<0.0001
N3	5.483	3.057–9.836	<0.0001	5.244	3.380–8.135	<0.0001
Grade			0.182			0.042*
Grade 1	1.000			1.000		
Grade 2	1.266	0.651–2.459	0.487	2.655	1.223–5.765	0.014
Grade 3	1.585	0.817–3.075	0.173	2.690	1.227–5.898	0.013
Menopause			0.977			0.633
Negative	1.000			1.000		
Positive	0.996	0.738–1.344		0.926	0.674–1.271	
