# Supplementary material for: Clinical Relevance of Liver Kinase B1(LKB1) Protein and Gene Expression in Breast Cancer
Source: Sci Rep. 2016 Feb 15;6:21374. doi: 10.1038/srep21374 (PMC4753425; doi:10.1038/srep21374)
Supplement: Supplementary Files [file srep21374-s1.pdf]

Clinical Relevance of Liver Kinase B1(LKB1) Protein and Gene Expression in Breast Cancer

I-Chun Chen[1, 2], M.D.; Yuan-Ching Chang[3], M.D., Ph.D.; Yen-Shen Lu[1, 4], M.D., Ph.D.; Kuei-Pin Chung[5], M.D.; Chiun-Sheng Huang[6], M.D., Ph.D.; Tzu-Pin Lu, Ph.D.[7]; Wen-Hung Kuo[6], M.D., Ph.D.; Ming-Yang Wang, M.D., Ph.D. [6]; Kuan-Ting Kuo[8], M.D., Ph.D.; Pei-Fang Wu[1, 9], M.D., Ph.D., Tsu-Hsin Hsueh[1], M.Sc.; Chen-Yang Shen, Ph.D[10],[11, 12] ;Ching-Hung Lin[1, 4, 13], M.D., Ph.D.*; Ann-Lii Cheng[1, 2, 4], M.D., Ph.D

1. Department of Oncology, National Taiwan University Hospital
2. Graduate Institute of Oncology, National Taiwan University
3. Department of Surgery, Mackay Memorial Hospital
4. Department of Internal Medicine, National Taiwan University Hospital
5. Departent of Laboratory Medicine, National Taiwan University Hospital
6. Department of Surgery, National Taiwan University Hospital
7. Department of Public Health, National Taiwan University
8. Department of Pathology, National Taiwan University Hospital
9. Department of Medical Research, National Taiwan University Hospital
10. Graduate Institute of Life Sciences, National Defense Medical Center, Taipei
11. Institute of Biomedical Sciences, Academia Sinica, Taipei, Taiwan
12. College of Public Health, China Medical University, Taichong, Taiwan
13. Oncology Center, National Taiwan University Hospital Hsin-Chu Branch

*Corresponding author:
Ching-Hung, Lin
Department of Oncology, National Taiwan University Hospital
No. 7, Chung-Shan South Road, Taipei, Taiwan, 10002
Oncology Center, National Taiwan University Hospital Hsin-Chu Branch, Hsin-Chu, Taiwan
No. 25, Lane 442, Section 1, Jingguo Rd, North District, Hsinchu City, 300
Phone: +886-2-23562859
Fax: +886-2-23711174
E-mail: chinghlin@ntu.edu.tw

Supplement Table 1. Demographics

	NTUH	MMH	METABRIC (Discovery)	METABRIC (Validation)
Patient Number	730	307	997	995
LKB1 Evaluable	600	295	997	995
Median Age	48.00	54.00	61.30	62.61
ER(+)	401(66.8%)	167(56.6%)	801(80.3%)	720(72.4%)
HER2(+)	135(22.5%)	87(30.0%)	117(11.7%)	132(13.2%)
LKB1(+)	425(70.8%)	205(69.5%)	498(49.9%)	497(50.1%)
Stage				
0	0(0%)	5(1.7%)	0(0%)	12(1.2%)
I	192(31%)	31(10.5%)	296(29.7%)	257(25.8%)
II	297(45%)	144(48.8%)	519(52.1%)	524(52.7%)
III	80(18%)	102(34.6%)	173(17.4%)	174(17.5%)
IV	26(4.3%)	13(4.4%)	9 (0.9%)	10(1.0%)
Unknown	5(1.2%)	0(0%)	0(0.0%)	18(1.8%)
Subtype				
IDC	693(94.9%)	290(94.5%)	794(79.6%)	759(76.3%)
IDC+ILC	0(0.0%)	0(0.0%)	46(4.6%)	44(4.4%)
ILC	28(3.8%)	5(1.6%)	68(6.8%)	80(8.0%)
Others*	9(1.2%)	12(3.9%)	89(8.9%)	112(11.2%)

*Unknown, DCIS, medullary carinoma, mucinous carcinoma, apocrine carcinoma, tubular carcinoma, invasive carcinoma, phyllodes tumor were included.

Supplement Table 2. Cox Regression Model：Relapse Free Survival

	NTUH			MMH			NTUH/MMH Summary Analysis		
	HR	95%CI	P	HR	95%CI	P	HR	95%CI	P
LKB1(IHC)			0.474			0.893			0.563
Negative	1.000			1.000			1.000		
Positive	1.176	0.754-1.833		1.032	0.653-1.631		1.097	0.801-1.503	
ER			0.022*			0.088			0.008*
Negative	1.000			1.000			1.000		
Positive	0.587	0.373-0.925		0.647	0.393-1.068		0.644	0.466-0.891	
HER2			0.243			0.166			0.093
Negative	1.000			1.000			1.000		
Positive	0.744	0.453-1.223		0.707	0.433-1.155		0.748	0.533-1.050	
T			0.004*			<0.0001*			<0.0001*
T1	1.000			1.000			1.000		
T2	1.562	0.976-2.499	0.063	0.824	0.472-1.438	0.495	1.265	0.886-1.807	0.196
T3	2.317	1.107-4.850	0.026	1.491	0.638-3.483	0.356	1.786	1.039-3.070	0.036
T4	4.691	1.892-11.629	0.001	5.045	2.134-11.930	<0.0001	5.521	3.028-10.069	<0.0001
N			0.007*			<0.0001*			<0.0001*
N0	1.000			1.000			1.000		
N1	1.679	1.071-2.633	0.024	1.998	1.106-3.611	0.022	1.852	1.302-2.636	0.001
N2	2.750	1.440-5.251	0.002	2.464	1.265-4.798	0.008	2.985	1.929-4.621	<0.0001
N3	2.332	1.002-5.426	0.049	4.542	2.401-8.593	<0.0001	4.533	2.903-7.078	<0.0001
Grade			0.148			0.300			0.014*
Grade 1	1.000			1.000			1.000		
Grade 2	1.764	0.918-3.391	0.089	3.104	0.724-13.320	0.127	2.166	1.208-3.885	0.010
Grade 3	2.050	0.990-4.243	0.053	3.213	0.728-14.188	0.123	2.527	1.359-4.698	0.003
Menopause			0.759			0.509			0.647
Negative	1.000			1.000			1.000		
Positive	0.938	0.625-1.409		0.855	0.538-1.360		0.933	0.691-1.258	

Supplement Table 3. LKB1, phosphor-AMPK, and phosphorylated ACC

	NTUH		
	LKB1 High	LKB1 Low	Total
Patient Number	58(53.7%)	50(46.3%)	108(100%)
pAMPK			
Negative	31(53.4%)	29(58.0%)	60(55.6%)
Positive	27(46.6%)	21 (42.0%)	48(44.4%)
p	0.700		
pACC			
Negative	26(56.6%)	40(58.3%)	66(61.1%)
Positive	32(43.4%)	10(41.6%)	42(38.9%)
p	0.0003		

Supplement Figure 1

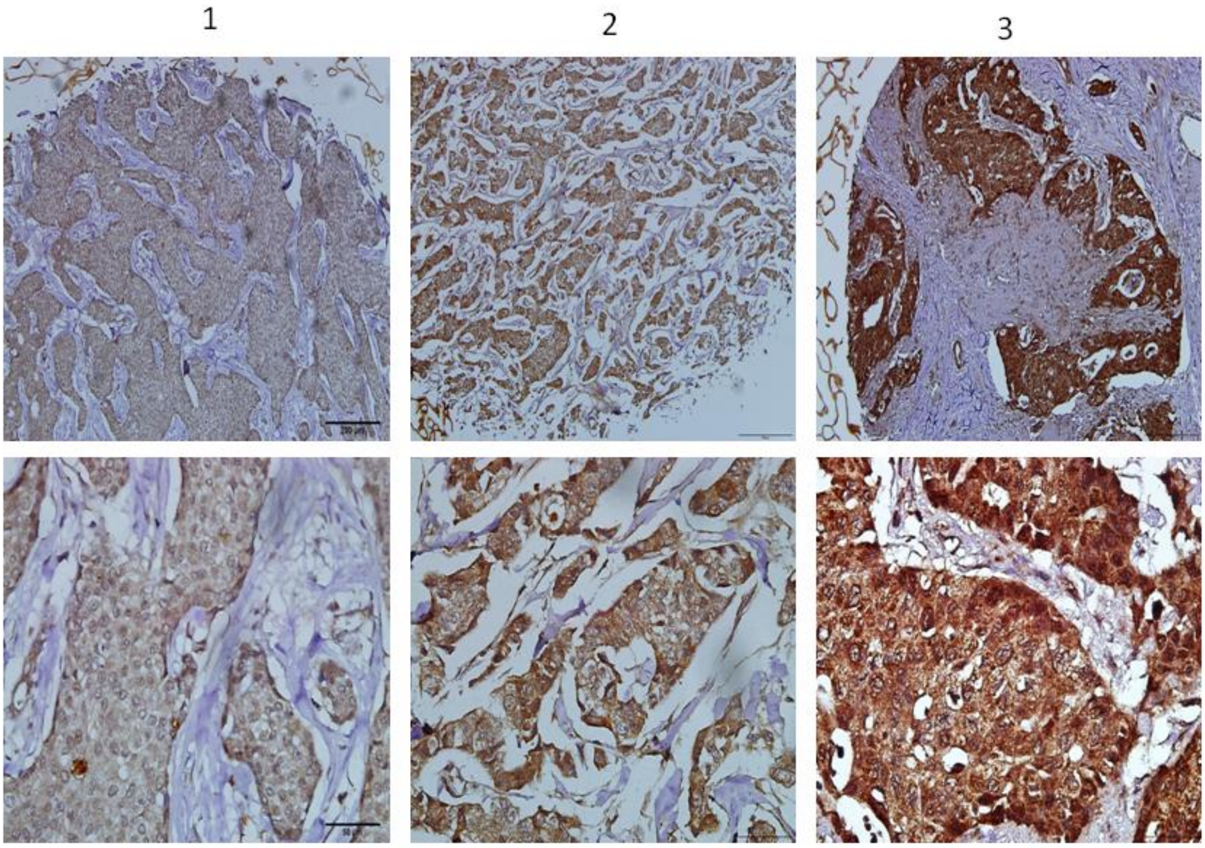

Supplementary Figure 1：LKB1 IHC stains：MMH cohort

IHC staining of LKB1 in tissue microarray slides from breast cancer patients in MMH cohort.

Supplement Figure 2

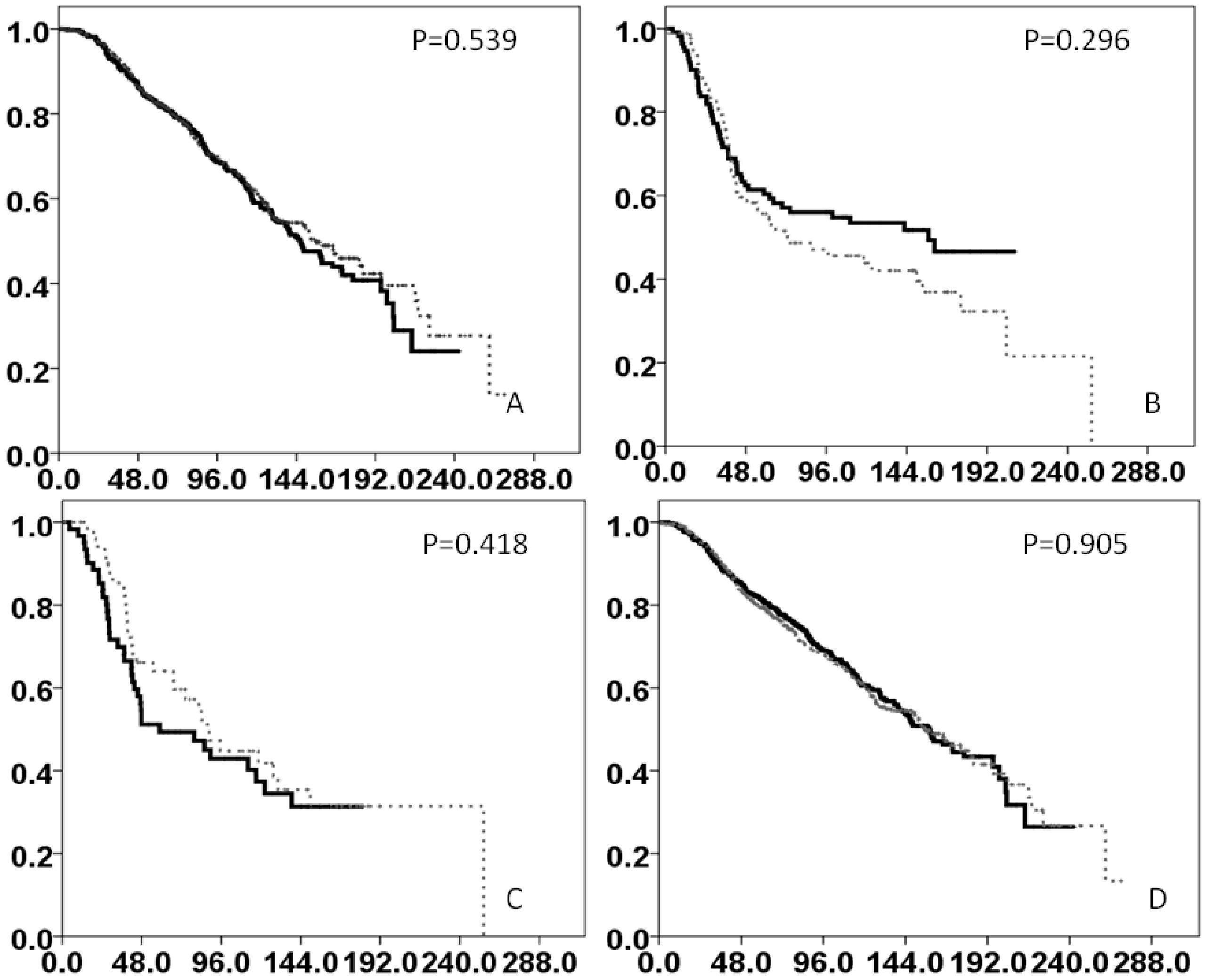

Supplementary Figure 2：OS (months) of METABRIC discovery cohort in subgroups. (A) ER positive (B)ER negative (C)HER2 positive (D) HER2 negative (Dotted line：LKB1 high, solid line：LKB1 low)

Supplement Figure 3

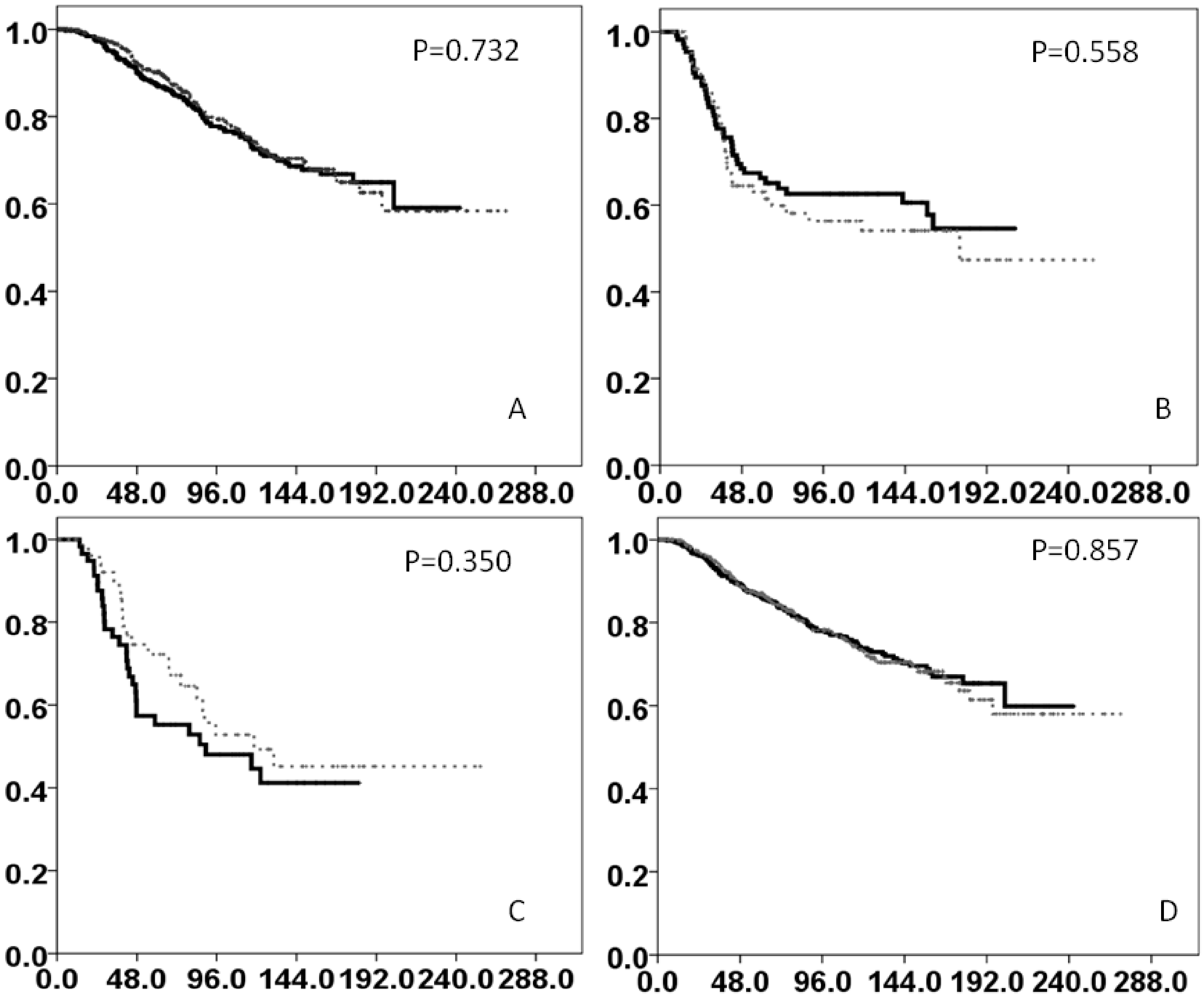

Supplementary Figure 3：OS (months) of METABRIC validation cohort in subgroups. (A) ER positive (B)ER negative (C)HER2 positive (D) HER2 negative (Dotted line：LKB1 high, solid line：LKB1 low)

Supplement Figure 4

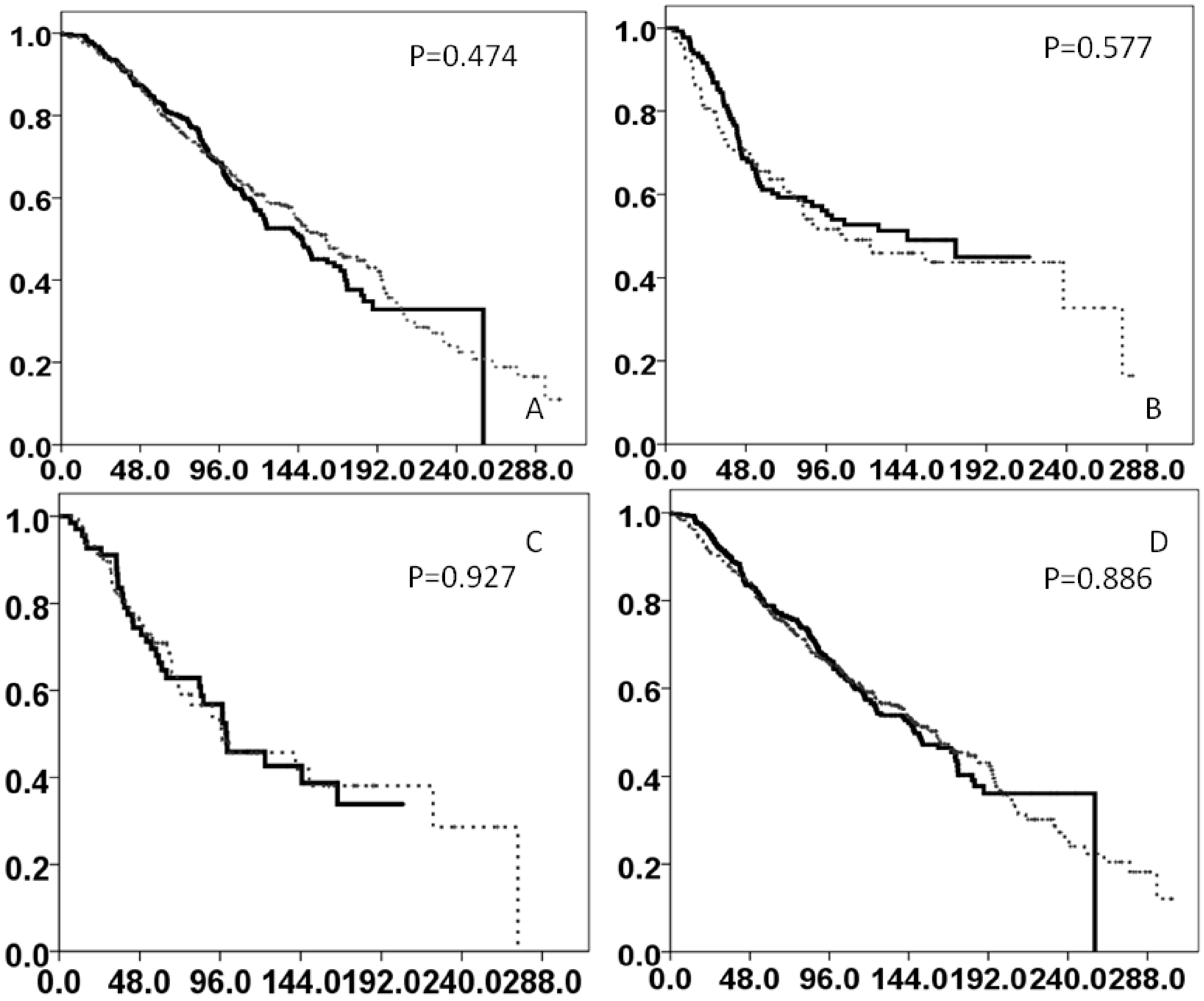

Supplementary Figure 4：BSS (months) of METABRIC discovery cohort in subgroups. (A) ER positive (B)ER negative (C)HER2 positive (D) HER2 negative (Dotted line：LKB1 high, solid line：LKB1 low)

Supplement Figure 5

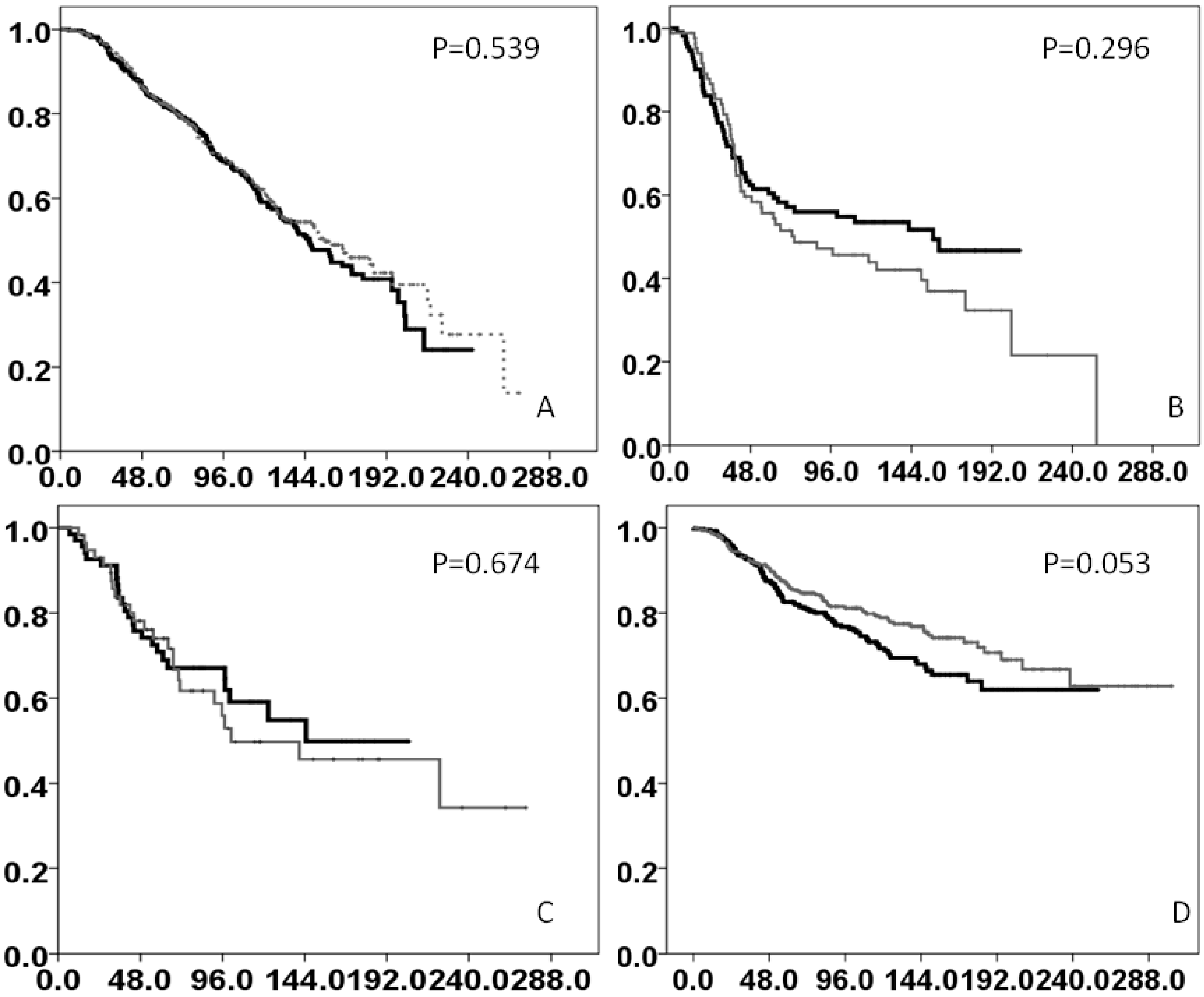

Supplementary Figure 5：BSS (months) of METABRIC validation cohort in subgroups. (A) ER positive (B)ER negative (C)HER2 positive (D) HER2 negative (Dotted line：LKB1 high, solid line：LKB1 low)

Supplement Figure 6

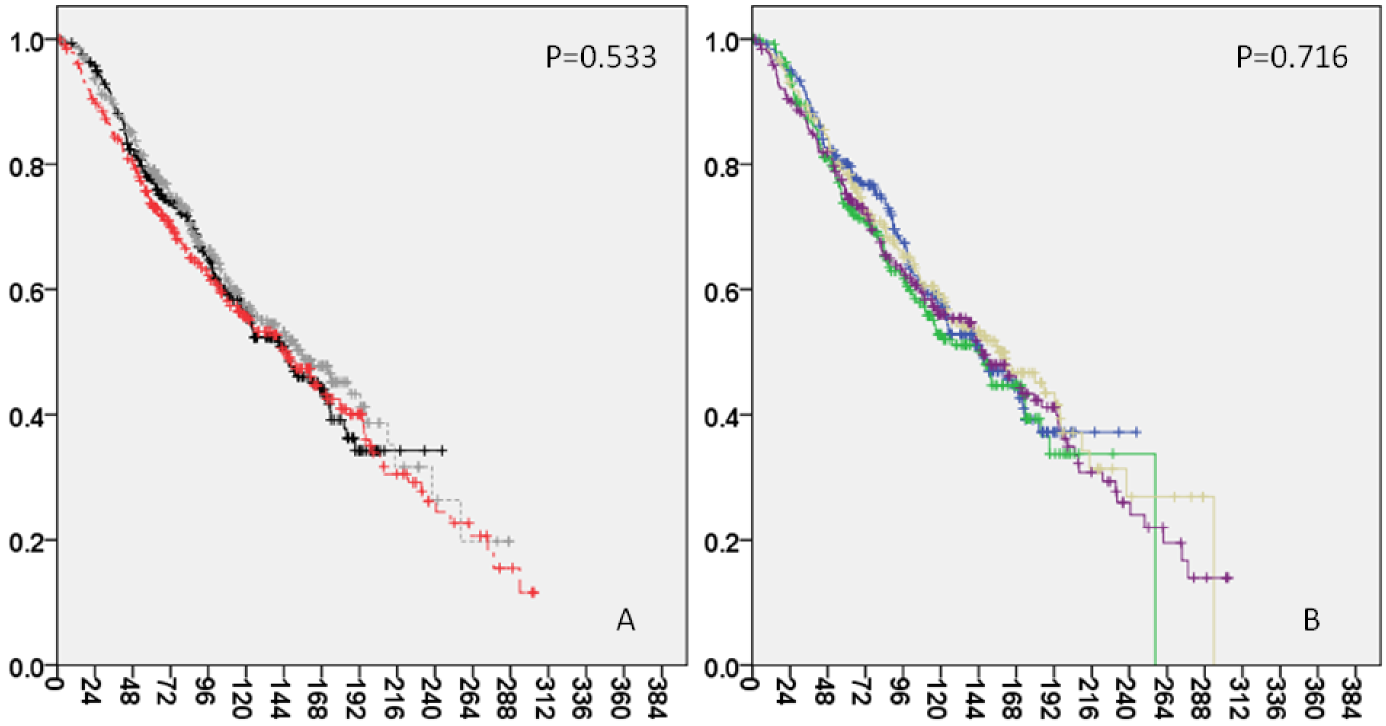

Supplementary Figure 6：

When *LKB1* mRNA expression level is grouped in thirds (A) or in quartile (B), the lack of prognostic value of LKB1 was persistently noted in the validation cohort.

Supplement Figure 7:

Phospho-AMPKα (Thr172), 200X

0 1 2 3

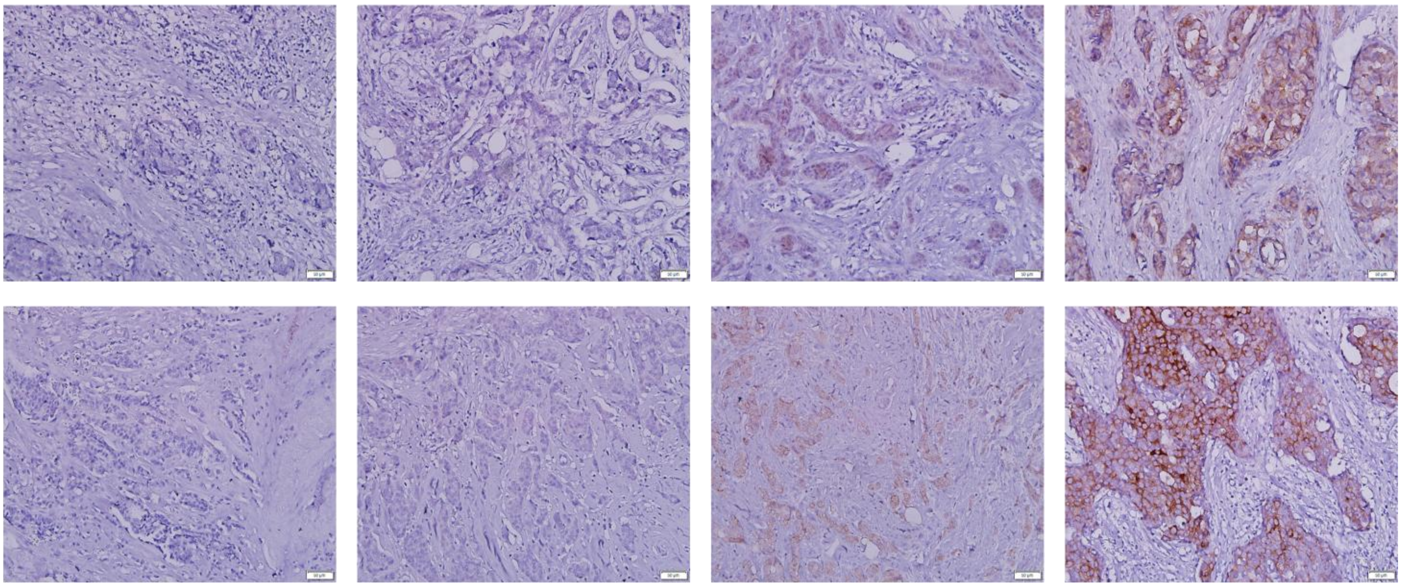

0 1 2 3

Phospho-Acetyl-CoA Carboxylase (Ser79), 200X

Supplement Figure 7:

Representative figures for pAMPK and pACC IHC staining slides from NTUH cohort.
